# Supplementary material for: Identification of Aging and Young Subtypes for Predicting Colorectal Cancer Prognosis and Immunotherapy Responses
Source: Int J Mol Sci. 2023 Jan 12;24(2):1516. doi: 10.3390/ijms24021516 (PMC9862647; doi:10.3390/ijms24021516)
Supplement: Supplementary file 1 [file ijms-24-01516-s001.zip › ijms-2133601-supplementary.pdf]

Identification of Aging and Young Subtypes for Predicting Colorectal Cancer Prognosis and Immunotherapy Responses

Lulu Tan[1,†] , Xiakeerzhati Xiaohalati[2,†], Feng Liu[3], Jia Liu[3], Haoyu Fu[1], Yang Zhang[2], Jinbo Gao[1], Kaixiong Tao[1], Guobin Wang[1], Lin Wang[2,*], Zheng Wang[1,*]

[1]*Department of Gastrointestinal Surgery, Union Hospital, Tongji Medical College, Huazhong University of Science and Technology, Wuhan, 430022, China*

[2]*Department of Clinical Laboratory, Union Hospital, Tongji Medical College, Huazhong University of Science and Technology, Wuhan, 430022, China*

[3]*Research Center for Tissue Engineering and Regenerative Medicine, Union Hospital, Tongji Medical College, Huazhong University of Science and Technology, Wuhan, 430022, China*

Corresponding author.

Lin Wang lin_wang@hust.edu.cn Department of Clinical Laboratory, Union Hospital, Tongji Medical College, Huazhong University of Science and Technology, Wuhan, 430022, China

Zheng Wang zhengwang@hust.edu.cn Department of Gastrointestinal Surgery, Union Hospital, Tongji Medical College, Huazhong University of Science and Technology, Wuhan, 430022, China

[†] These authors contributed equally to this work.

KEYWORDS: colorectal cancer; aging; unsupervised clustering; tumor microenvironment; prognosis

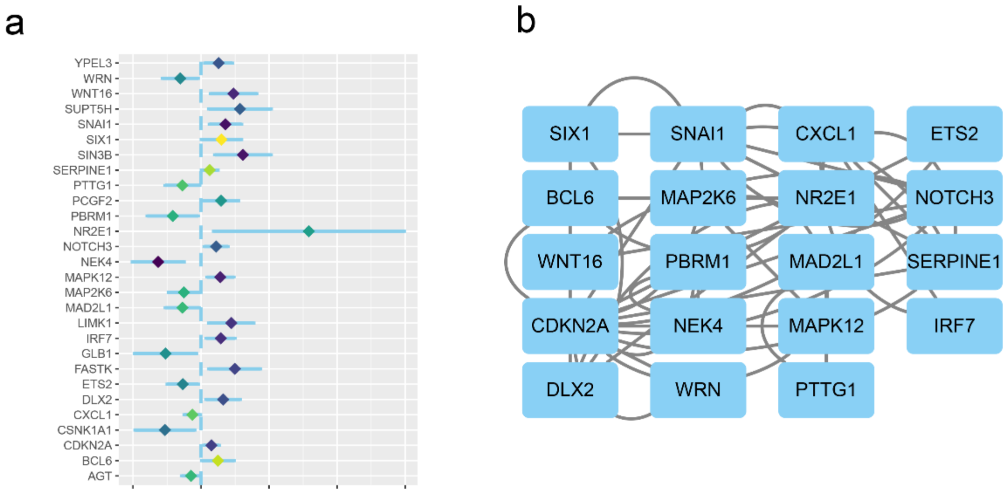

Figure S1. Twenty-eight aging-related genes were associated with CRC prognosis. (**a**) Univariate cox regression analyses of 28 aging-related genes. (**b**) PPI networks of aging-related genes.

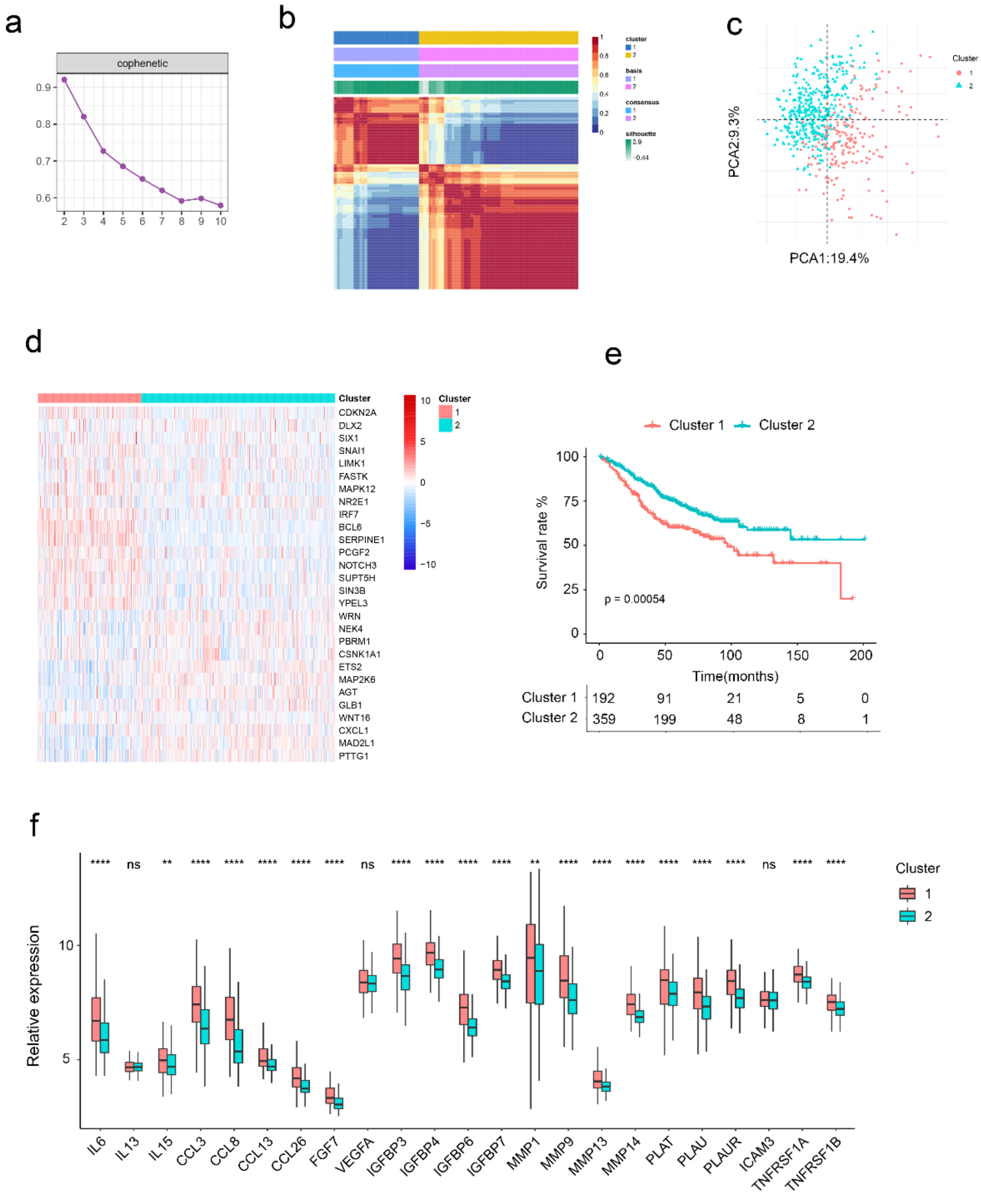

Figure S2. Unsupervised clustering of aging-related genes in the validation set. (**a**,**b**) The validation set (GSE39582) was clustered by NMF method. (**c**) Visualization of NMF results using PCA. (**d**) Heatmap of 28 aging-related genes. (**e**) Kaplan–Meier curves for OS of GSE39582 with the aging classes. (**f**) Gene expression of the SASP gene sets between two distinct clusters in GSE39582. **, $P < 0.01$; ****, $P < 0.0001$; ns, not significant.

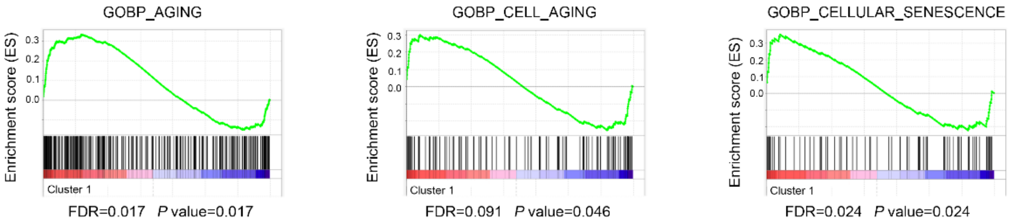

Figure S3. GSEA analyzed the aging biological pathways of two aging subtypes.

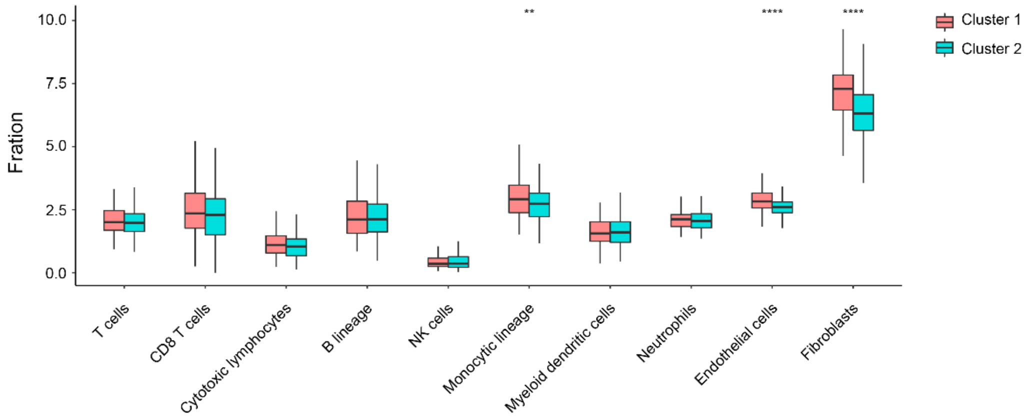

Figure S4. Box plots of fractions of 10 immune cells by MCP-counter algorithm. **, $P < 0.01$; ****, $P < 0.0001$.

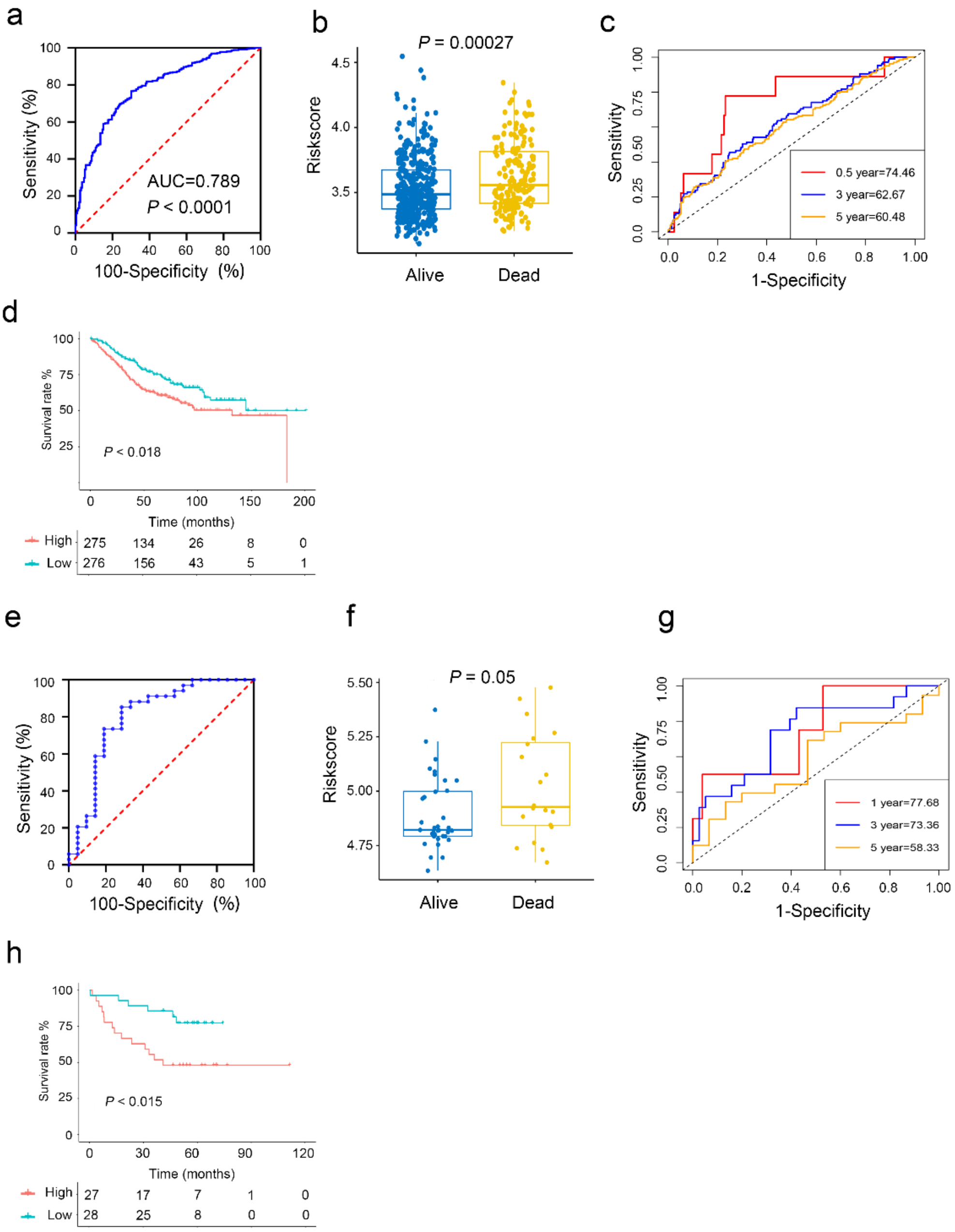

Figure S5. Validation of aging-related risk score. (**a**,**e**) ROC curves for predicting aging subtypes by risk score in the validation sets (GSE39582, GSE17437). (**b**,**f**) Comparison of risk scores in survival and death patients. (**c**,**g**) ROC curves for predicting 3-, and 5-year OS by risk score in the validation sets (GSE39582, GSE17437). (**d**,**h**) The association of high-risk score with prognosis of CRC patients.

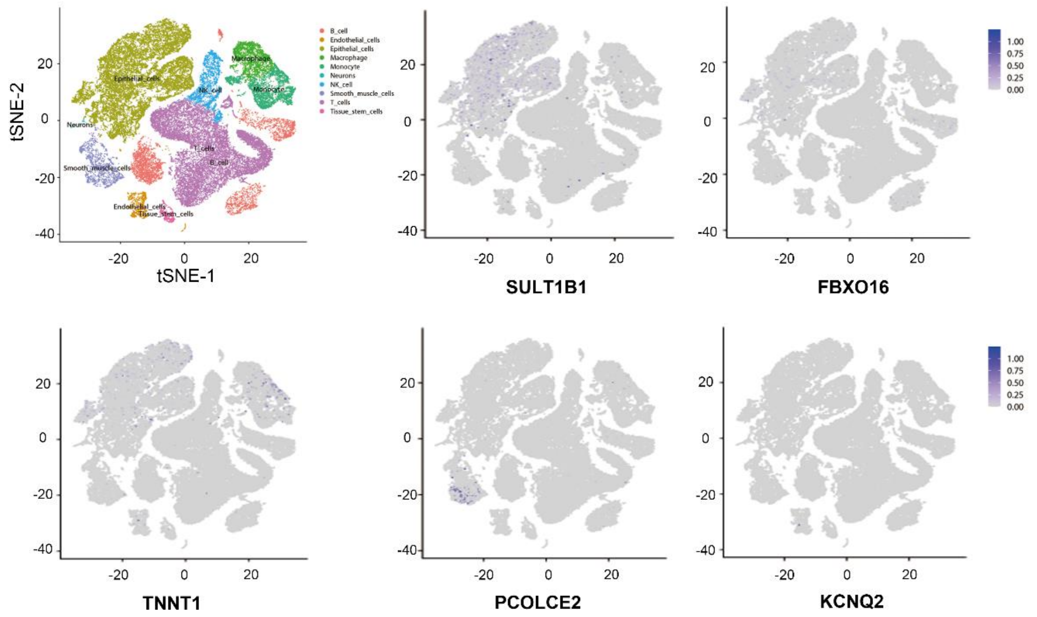

Figure S6. t-SNE plot showed single-cell transcriptomic profiling of SULT1B1, FBXO16, TNNT1, PCOLCE2 and KCNQ2 from CRC.

Table. S1. List of the primer sequences

Primer name	Primer sequence (from 5’ to 3’)
KCNQ2 Forward	ATCCTGGAAATCGTGACTATCGT
KCNQ2 Reverse	TTCCGGGCAAACTTGAGCC
NOG Forward	CCATGCCGAGCGAGATCAAA
NOG Reverse	TCGGAAATGATGGGGTACTGG
PCOLCE2 Forward	TACTTGGAAAATCACAGTTCCCG
PCOLCE2 Reverse	CGGCACAGGTTGTCACTCTC
NKAIN4 Forward	GTCGTCCTCTGCGCTTTTCA
NKAIN4 Reverse	CCGAAGAGTCCCAGGATGAC
HOXC6 Forward	ACAGACCTCAATCGCTCAGGA
HOXC6 Reverse	AGGGGTAAATCTGGATACTGGC
HOTAIR Forward	CAGTGGGGAACTCTGACTCG
HOTAIR Reverse	GTGCCTGGTGCTCTCTTACC
HOXC8 Forward	ACCGGCCTATTACGACTGC
HOXC8 Reverse	TGCTGGTAGCCTGAGTTGGA
PANX2 Forward	TTCGCCGCCATCATGTACG
PANX2 Reverse	GGTAACAGTTGTCGATCTCCTG
HOXC11 Forward	ATGTTTAACTCGGTCAACCTGG
HOXC11 Reverse	GCATGTAGTAAGTGCAACTGGG

TNNT1 Forward	ACATTTCGAGCAGCGGAAGA
TNNT1 Reverse	CTTCTCCTCCGCCAGCTTAG
MUC16 Forward	CCAGTCCTACATCTTCGGTTGT
MUC16 Reverse	AGGGTAGTTCCTAGAGGGAGTT
CLCA1 Forward	ACAACAATGGCTATGAAGGCA
CLCA1 Reverse	GGTCTCAAGTTTTGGTCTCACAT
SULT1B1 Forward	TTGAACAGTTCCATAGCAGACC
SULT1B1 Reverse	CAGGGAGAGTCATTTCCAACATT
FBXO16 Forward	AGGATGGATTTGTAATCGCTGAC
FBXO16 Reverse	CGAAAAGCTGATAAAGGGGACT
P21 Forward	TGTCCGTCAGAACCCATGC
P21 Reverse	AAAGTCGAAGTTCCATCGCTC
MMP-1 Forward	AAAATTACACGCCAGATTTGCC
MMP-1 Reverse	GGTGTGACATTACTCCAGAGTTG
CCL2 Forward	CAGCCAGATGCAATCAATGCC
CCL2 Reverse	TGGAATCCTGAACCCACTTCT
LMNB1 Forward	GAAAAAGACAACTCTCGTCGCA
LMNB1 Reverse	GTAAGCACTGATTTCCATGTCCA
GAPDH Forward	TGCACCACCAACTGCTTAGC
GAPDH Reverse	GGCATGGACTGTGGTCATGAG
